# Toxicity and Oncologic Outcomes of Proton Radiotherapy for Oropharyngeal Cancer: A Systematic Review and Meta-Analysis

**DOI:** 10.7759/cureus.78849

**Published:** 2025-02-11

**Authors:** Niema B Razavian, Rachel F Shenker, Sydney Smith, Ralph B D'Agostino, Ryan T Hughes

**Affiliations:** 1 Radiation Oncology, Moffitt Cancer Center, Tampa, USA; 2 Radiation Oncology, Intermountain Health, Salt Lake City, USA; 3 Biostatistics, Wake Forest School of Medicine, Winston-Salem, USA; 4 Radiation Oncology, Wake Forest School of Medicine, Winston-Salem, USA

**Keywords:** intensity-modulated radiotherapy, oropharynx cancer, patient reported health outcomes, proton beam radiotherapy, systematic review and meta-analysis

## Abstract

Intensity-modulated radiotherapy (IMRT) for oropharyngeal cancer (OPC) is associated with acute and late toxicities that impact patient quality of life. Proton radiotherapy (PRT) can reduce exposure to surrounding tissues, but the clinical magnitude of this advantage is unclear. A systematic review and meta-analysis was performed in accordance with the Preferred Reporting Items for Systematic Reviews and Meta-Analyses (PRISMA) guidelines. Included studies reported toxicity or oncologic outcomes from patients treated with PRT for OPC. Pooled outcomes were estimated using random-effects models. Comparisons between PRT and IMRT were performed using log odds ratios. Primary outcomes were the pooled rates of adverse events, overall survival (OS), and progression-free survival (PFS). A total of 18 studies (16 retrospective, two prospective) consisting of 956 patients were identified. Pooled rates of acute grade 3+ toxicities were as follows: dermatitis 19%, mucositis 32%, xerostomia 1.3%, dysphagia 13%, and weight loss 1.4%. The pooled rate of acute hospitalizations was 10%. Among studies reporting late toxicities, the rates of grade 3+ xerostomia and dysphagia were 1.1% and 1.6%, respectively. Compared to IMRT, PRT was associated with lower rates of acute feeding tube use (21% versus 31%; P = 0.0012), but not long-term feeding tube use (1.4% versus 2.7%; P = 0.24). After PRT, OS at two and three years were 98% and 96%, while PFS at two and three years were 93% and 86%. PRT for patients with OPC is associated with favorable toxicity and oncologic outcomes. While randomized clinical trials are ongoing, these data provide additional evidence regarding the efficacy of PRT in the upfront treatment of OPC.

## Introduction and background

Proton radiotherapy (PRT) is an emerging treatment modality for patients with oropharyngeal cancer (OPC) [1,2]. Compared to intensity-modulated radiotherapy (IMRT), PRT has distinct physical properties that allow for more conformal dose distributions and lower doses to surrounding normal tissues [1,3]. While PRT is purported to improve the side effect profile associated with radiotherapy (RT) for OPC, data are primarily limited to single-institution series [4,5], its potential for increased biologic effectiveness may increase the risk of late toxicity [6], and randomized, comparative trials with IMRT are ongoing [7,8]. Given the potential increases in cost and resource utilization [8-10], a clearer understanding of outcomes associated with PRT would strongly inform clinical practice and future resource allocation. 

We performed a systematic review and meta-analysis to better understand the safety and efficacy of PRT for patients with OPCs. The primary objective of this study was to determine the rates of toxicity and oncologic outcomes after PRT. Additionally, patient-reported outcomes (PROs), patterns of failure, and comparisons between PRT and IMRT were assessed. An improved understanding of PRT will allow for more comprehensive discussions in an area where the optimal balance of risk and benefit is unclear. 

## Review

Methods

Search Strategy

A systematic literature review was performed following the Preferred Reporting Items for Systematic Reviews and Meta-Analyses (PRISMA) guidelines [11]. Review and analysis were designed prospectively and registered with the International Prospective Register of Systematic Reviews (PROSPERO) (CRD42023444520). 

Four electronic databases (PubMed, Embase, Web of Science, and Cochrane Library) were queried for published articles from 01/01/1980 to 05/01/2024. Searches were performed on 05/01/24.

The following search terms were used to query each database: ("Tonsil" OR "tonsillar" OR "tonsils" OR "oropharynx" OR "oropharyngeal" or "base of tongue" or “BOT” or “soft palate”) AND ("Neoplasm" OR "neoplasms" OR "carcinoma" OR "carcinomas" OR "Cancer" OR "Cancers") AND ("radiotherapy" OR "Radiotherapies" OR "Radiation Therapy" OR "Targeted Radiation Therapies" OR "radiation" OR "RT" OR "irradiation" OR "IMRT") AND ("proton" OR "protons" OR "IMPT" OR "PBS" OR "intensity-modulated proton therapy" OR "intensity modulated proton therapy" OR "pencil beam scanning" OR "passive scattering").

After removing duplicate publications, titles and abstracts were screened by at least two reviewers. In the case of discordance, a third reviewer was added, and inclusion was determined by consensus opinion. Articles were included for analysis if they contained at least 10 patients with OPCs treated with PRT in the upfront setting and reported oncologic outcomes (progression-free survival (PFS) or overall survival (OS)), clinician-rated toxicity outcomes, or PROs. Studies that used IMRT alone, IMRT combined with PRT, or PRT for re-irradiation were excluded. 

*Full Inclusion and Exclusion Criteria (Population, Intervention, Comparator, Outcomes, Timing, and Setting (PICOTS) Format)* 

Population: Included peer-reviewed studies treated 10 or more patients with squamous cell carcinoma of the oropharynx (e.g., tonsil, base of tongue). Patients of all genders, race, socioeconomic status, and comorbidities were eligible for inclusion. Studies treating patients with other head and neck tumor sites (e.g., oral cavity, larynx) were excluded.

Intervention: Included studies treated patients with PRT in the upfront setting. Studies that used only IMRT, PRT as a boost to IMRT, and PRT in the setting of re-irradiation or re-treatment were excluded.

Comparator: This study was designed to describe oncologic outcomes and adverse events associated with PRT; thus, no control group was specified for the primary endpoint. However, for studies that reported outcomes of IMRT, this was considered the comparator treatment for secondary analysis (see below).

Outcomes: The primary outcome was the pooled rates of adverse events (both acute and late), OS, and PFS after PRT. Secondary outcomes included PRO measures and a comparison of outcomes between PRT and IMRT. Pooled rates were estimated using a random-effects model.

Timing: Included studies treated patients with radiation in either a definitive or adjuvant (post-operative) manner. Patient follow-up was performed per the institutional standard of care.

Setting: Included studies consisted of patients treated by radiation oncologists, which is generally delivered in the outpatient setting. 

Statistical Methods

Primary outcomes were the pooled rates of acute or late clinician-rated toxicities, OS, and PFS following PRT. Secondary outcomes included PROs and comparisons of outcomes between PRT and IMRT. Pooled rates were estimated using a random-effects model for outcomes reported in three or more studies. To reduce confounding, if studies had overlapping patient populations, pooled analysis of a given outcome was performed using only the study with the larger population. Heterogeneity among the studies was assessed using the Higgins I2 test. If heterogeneity was moderate or high (I2 > 50%), outlier studies were identified using Cook's distance and removed, and then pooled rates were re-estimated using random-effects models. Differences in rates of outcomes between PRT and IMRT were assessed using a random-effects meta-analysis comparing log odds ratios (ORs) across studies. For this analysis, only studies that reported patients treated with PRT or IMRT were included. The quality of studies included for statistical analysis was quantified using the Methodological Index for Non-randomized Studies (MINORS) criteria [12], and publication bias was assessed using Egger's test for funnel plot asymmetry. Statistical significance was defined as P < 0.05. All tests were performed using the "metafor" package (Version 4.6-0) in R version 4.4.1 (R Foundation for Statistical Computing, Vienna, Austria (https://www.R-project.org/)).

Results

Characteristics of the Included Studies

**Figure 1:**
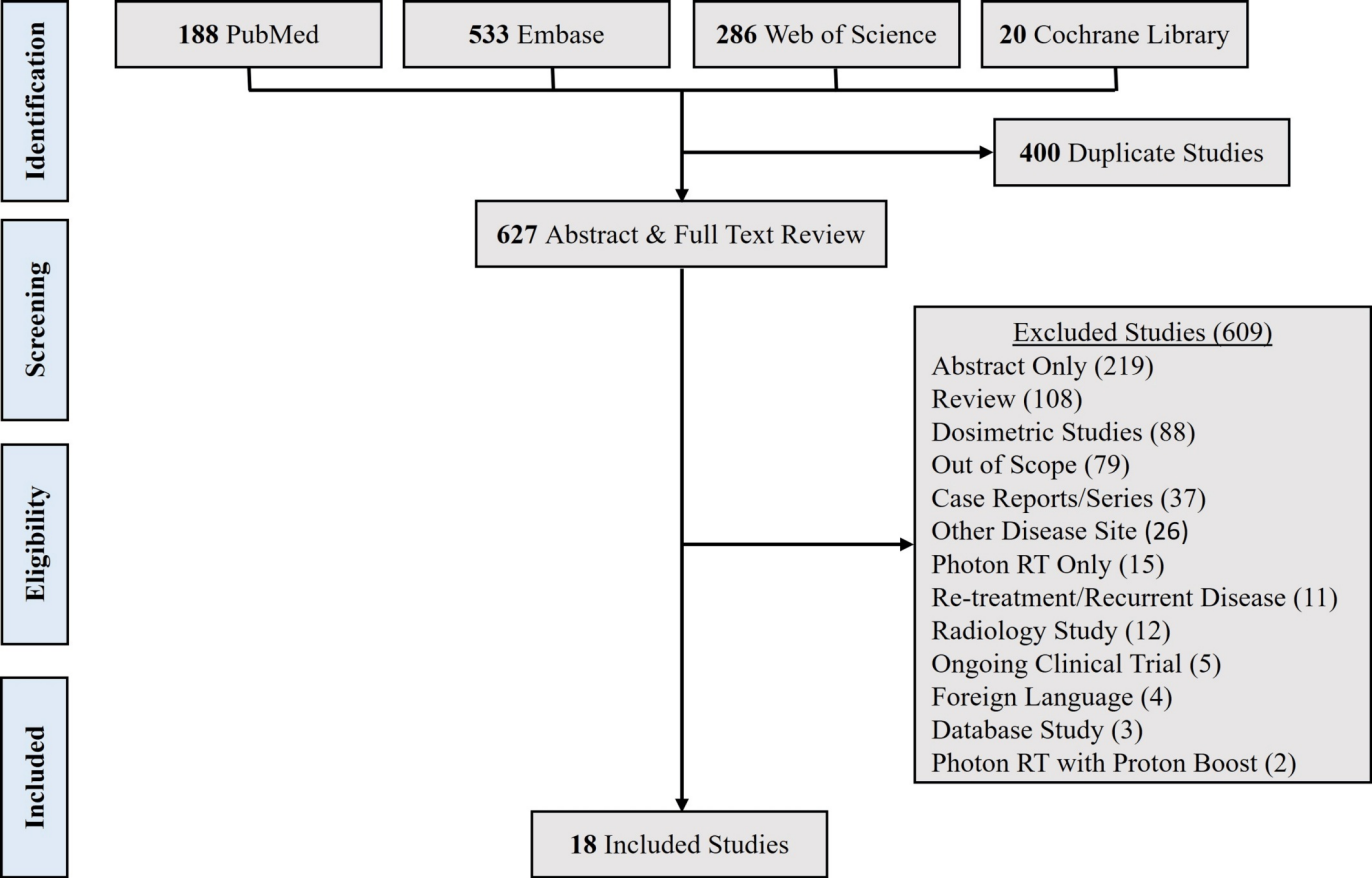
PRISMA diagram PRISMA: Preferred Reporting Items for Systematic Reviews and Meta-Analyses; RT: radiotherapy

After removing duplicate citations, 627 abstracts were screened, and 18 articles (956 patients) underwent full-text review and analysis (Figure 1) [4,5,13-26]. Baseline characteristics, treatments, and outcomes from the included studies are summarized in Table 1 and Table 2. Published from 2016 to 2023, most studies (16 of 18) were retrospective and had a median follow-up ranging from 7.7 to 40.6 months. The median study quality was 16 (Table 3) and publication bias was not identified. All studies used pencil beam scanning PRT, and eight studies reported a separate population of patients treated with IMRT. Among patients receiving PRT, treatment was delivered in either the definitive (14 studies) or post-operative (eight studies) setting. The median age ranged from 55.5 to 69.1 years old. The American Joint Committee on Cancer (AJCC) 7th Edition was the most frequently employed staging system: in 15 studies, the majority of patients had T1-2, N2-3, and stage IV disease. Among the 14 studies reporting human papillomavirus (HPV) status, 70-100% of patients had HPV-associated disease. Use of concurrent chemotherapy ranged from 23% to 100% (15 studies).

**Table 1:** Clinical and treatment characteristics of patients treated with proton RT R: retrospective; P: prospective; MDACC: MD Anderson Cancer Center; UW: University of Washington; MSKCC: Memorial Sloan Kettering Cancer Center; N/A: not available; FU: follow-up; HPV: human papillomavirus; RT: radiotherapy; BOT: base of tongue; AJCC: American Joint Committee on Cancer; UPenn: University of Pennsylvania

Author (citation)	Study type	Study period	Institution	Total	Median FU, months (range)	Median age, years (range)	Men	Women	Any smoking	No smoking	Smoking unknown	HPV positive	HPV negative or unknown	BOT primary	Tonsil/other primary	Staging edition	Tumor (T)-stage	Nodal (N)-stage	Group stage	Definitive RT	Adjuvant RT	Median RT dose, Gy (range)	Induction chemotherapy	Concurrent chemotherapy	No chemotherapy	Acute feeding tube use	Late feeding tube use
Blanchard et al. [13]	R	2010-2014	MDACC	50	29 (8-49)	61 (37-84)	42 (84%)	8 (16%)	25 (50%)	25 (50%)	N/A	44 (88%)	6 (12%)	23 (46%)	27 (54%)	N/A	T1-2: 40 (80%), T3-4: 10 (20%)	N0-1: 10 (20%), N2-3: 40 (80%)	I-III: 10 (20%), IV: 40 (80%)	50 (100%)	0 (0%)	N/A (N/A)	20 (40%)	32 (46%)	N/A	12 (24%)	1 (2%)
Gunn et al. [4]	P	2011-2014	MDACC	50	29 (8-49)	61 (37-84)	42 (84%)	8 (16%)	25 (50%)	25 (50%)	N/A	44 (88%)	6 (12%)	21 (42%)	29 (58%)	N/A	T1-2: 40 (80%), T3-4: 10 (20%)	N0-1: 10 (20%), N2-3: 40 (80%)	I-III: 10 (20%), IV: 40 (80%)	49 (98%)	1 (2%)	70 (60-70)	20 (40%)	32 (46%)	13 (26%)	N/A	N/A
Sio et al. [14]	R	2012-2015	MDACC	35	7.7 (N/A)	69.1 (N/A)	30 (86%)	5 (14%)	N/A	N/A	N/A	26 (74%)	9 (26%)	20 (57%)	15 (43%)	N/A	T1-2: 31 (89%), T3-4: 4 (11%)	N0-2a: 15 (42%), N2b-3: 20 (58%)	I-III: 11 (31%), IV: 24 (69%)	35 (100%)	0 (0%)	70 (59-70)	26 (74%)	35 (100%)	0 (0%)	N/A	N/A
Jensen et al. [15]	R	2010-2014	MDACC	50	N/A (N/A)	N/A (N/A)	N/A	N/A	N/A	N/A	N/A	N/A	N/A	N/A	N/A	N/A	N/A	N/A	N/A	50 (100%)	0 (%)	N/A (N/A)	N/A	N/A	N/A	N/A	N/A
Zhang et al. [16]	R	2011-2014	MDACC	50	34.6 (N/A)	N/A (N/A)	42 (84%)	8 (16%)	N/A	N/A	N/A	35 (70%)	15 (30%)	21 (42%)	29 (58%)	N/A	T1-2: 40 (80%), T3-4: 10 (20%)	N0-1: 10 (20%), N2-3: 40 (80%)	N/A	50 (100%)	0 (0%)	N/A (N/A)	20 (40%)	32 (64%)	N/A	N/A	N/A
Sharma et al. [17]	R	2013-2015	UPenn	31	N/A (N/A)	60 (N/A)	27 (87%)	4 (13%)	N/A	N/A	N/A	N/A	N/A	11 (36%)	21 (64%)	N/A	T1-2: 28 (90%), T3-4: 3 (10%)	N0-2b: 31 (100%), N2c-3: 0 (0%)	I-III: 4 (13%), IV: 27 (87%)	0 (0%)	31 (100%)	62.6 (N/A)	N/A	12 (39%)	19 (61%)	N/A	0 (0%)
Aljabab et al. [18]	R	2015-2017	UW	46	19.2 (N/A)	58 (N/A)	43 (93%)	3 (7%)	15 (33%)	31 (67%)	N/A	41 (89%)	5 (11%)	19 (41%)	27 (59%)	AJCC 7th	N/A	N/A	I-III: 5 (11%), IV: 41 (89%)	29 (63%)	17 (37%)	70 (60-74.4)	0 (0%)	31 (67%)	15 (33%)	18 (39%)	1 (2.2%)
Rwigema et al. [19]	R	2013-2016	UPenn	30	N/A (N/A)	58.2 (N/A)	26 (87%)	4 (13%)	N/A	N/A	N/A	N/A	N/A	N/A	N/A	N/A	T1-2: 25 (83%), T3-4: 5 (17%)	N0: 1 (3%), N1-3: 29 (97%)	N/A	0 (0%)	30 (100%)	62.2 (N/A)	N/A	7 (23%)	23 (77%)	N/A	N/A
Bagley et al. [20]	R	2012-2016	MDACC	69	N/A (N/A)	64 (37-84)	60 (87%)	9 (13%)	38 (55%)	31 (45%)	N/A	58 (84%)	11 (16%)	37 (54%)	32 (46%)	AJCC 7th	T1-2: 54 (78%), T3-4: 15 (22%)	N0-1: 16 (19%), N2-3: 52 (81%)	I-III: 14 (20%), IV: 55 (80%)	69 (100%)	0 (0%)	69.3 (60-70)	16 (23%)	49 (71%)	15 (22%)	N/A	N/A
Grant et al. [21]	R	2012-2017	MDACC	71	N/A (N/A)	63 (37-84)	62 (87%)	9 (13%)	37 (52%)	30 (42%)	4 (6%)	61 (86%)	10 (14%)	35 (49%)	36 (51%)	AJCC 7th	T1-2: 56 (79%), T3-4: 15 (21%)	N0-1: 15 (21%), N2-3: 56 (79%)	I-III: 13 (18%), IV: 58 (82%)	71 (100%)	0 (0%)	69.3 (52.5-70.1)	15 (21%)	51 (72%)	15 (21%)	N/A	N/A
Manzar et al. [22]	R	2013-2018	Mayo	46	12 (N/A)	66 (40-79)	43 (93%)	3 (7%)	27 (58%)	19 (40%)	1 (2%)	39 (85%)	7 (15%)	21 (46%)	25 (54%)	AJCC 7th	T1-2: 19 (41%), T3-4: 24 (52%)	N0-1: 10 (22%), N2-3: 35 (76%)	I-III: 7 (15%), IV: 36 (78%)	27 (59%)	19 (41%)	70 (56-70)	N/A	36 (78%)	10 (22%)	17 (41%)	N/A
Bahig et al. [23]	R	2011-2018	MDACC	57	33.6 (N/A)	60 (41-84)	49 (86%)	8 (14%)	29 (51%)	28 (49%)	N/A	52 (91%)	5 (8%)	N/A	N/A	AJCC 7th	T1-2: 45 (79%), T3-4: 12 (21%)	N0-1: 11 (19%), N2-3: 46 (81%)	I-III: 9 (16%), IV: 36 (78%)	53 (93%)	4 (7%)	N/A (N/A)	16 (28%)	39 (68%)	13 (23%)	N/A	N/A
Cao et al. [24]	R	2011-2015	MDACC	103	37.7 (N/A)	60 (33-85)	90 (87%)	13 (13%)	N/A	N/A	N/A	79 (77%)	24 (23%)	53 (52%)	50 (48%)	N/A	T1-2: 67 (65%), T3-4: 36 (35%)	N0-1: 24 (23%), N2-3: 79 (77%)	N/A	103 (100%)	0 (0%)	N/A (N/A)	30 (29%)	79 (76%)	N/A	N/A	N/A
Wright et al. [25]	R	2015-2019	UPenn	53	20.4 (6-50)	62 (47-77)	N/A	N/A	N/A	N/A	N/A	53 (100%)	0 (0%)	19 (36%)	34 (64%)	N/A	T1-2: 40 (76%), T3-4: 10 (19%)	N0-1: 43 (81%), N2-3: 10 (19%)	I-III: 53 (100%), IV: 0 (0%)	0 (0%)	53 (100%)	60 (60-63)	N/A	16 (30%)	37 (70%)	N/A	N/A
Anderson et al. [5]	P	2016-2019	Mayo	44	N/A (N/A)	N/A (N/A)	N/A	N/A	N/A	N/A	N/A	44 (100%)	0 (0%)	N/A	N/A	AJCC 7th	T1-2: 44 (100%), T3-4: 0 (0%)	N/A	N/A	0 (0%)	44 (100%)	N/A (N/A)	N/A	N/A	N/A	0 (0%)	N/A
Youssef et al. [26]	R	2018-2021	MSKCC	58	20 (N/A)	65.2 (N/A)	50 (86%)	8 (14%)	24 (41%)	34 (59%)	N/A	57 (98%)	1 (2%)	N/A	N/A	N/A	T1-2: 34 (59%), T3-4: 24 (41%)	N0-1: 13 (22%), N2-3: 45 (78%)	N/A	58 (100%)	0 (0%)	Primary: 70 (N/A), neck: 30 (N/A)	N/A	55 (95%)	3 (5%)	4 (6.9%)	0 (0%)
Pollock et al. [27]	R	2016-2021	Maryland	60	17 (N/A)	64 (44-84)	56 (93%)	4 (7%)	37 (62%)	23 (38%)	N/A	60 (100%)	0 (0%)	31 (52%)	29 (48%)	AJCC 7th	T1-2: 38 (63%), T3-4: 22 (37%)	N0-1: 30 (50%), N2-3: 30 (50%)	N/A	60 (100%)	0 (0%)	70 (56-70)	N/A	60 (100%)	0 (0%)	N/A	N/A
Singh et al. [6]	R	2013-2019	MSKCC	53	N/A (N/A)	N/A (N/A)	N/A (N/A)	N/A (N/A)	N/A (N/A)	N/A (N/A)	N/A (N/A)	N/A (N/A)	N/A (N/A)	N/A	N/A	N/A	N/A	N/A	N/A	N/A	N/A	N/A	N/A	N/A	N/A	N/A	N/A

**Table 2:** Clinical and treatment characteristics of patients treated with IMRT IMRT: intensity-modulated radiotherapy; R: retrospective; P: prospective; MDACC: MD Anderson Cancer Center; MSKCC: Memorial Sloan Kettering Cancer Center; N/A: not available; FU: follow-up, HPV: human papillomavirus; RT: radiotherapy; BOT: base of tongue; UPenn: University of Pennsylvania

Author (citation)	Study type	Treatment period	Institution	Total	Median FU, months (range)	Median age, years (range)	Men	Women	Any smoking	No smoking	Unknown smoking	HPV positive	HPV negative or unknown	BOT primary	Tonsil/other primary	Tumor (T)-stage	Nodal (N)-stage	Group stage	Definitive RT	Adjuvant RT	Median RT dose, Gy (range)	Induction chemotherapy	Concurrent chemotherapy	No chemotherapy	Acute feeding tube use	Late feeding tube use
Blanchard et al. [13]	R	2010-2012	MDACC	100	33 (2-55)	55.5 (34-78)	86 (86%)	14 (14%)	55 (55%)	45 (45%)	N/A	87 (87%)	13 (13%)	46 (46%)	54 (54%)	T1-2: 80 (80%), T3-4: 20 (20%)	N0-1: 20 (20%), N2-3: 80 (80%)	I-III: 20 (20%), IV: 80 (80%)	100 (100%)	0 (0%)	N/A (N/A)	44 (44%)	64 (64%)	N/A	38 (38%)	7 (7%)
Sio et al. [14]	R	2006-2014	MDACC	46	2.7 (N/A)	57.2 (N/A)	42 (91%)	4 (9%)	N/A	N/A	N/A	6 (13%)	40 (87%)	23 (50%)	23 (50%)	T1-2: 28 (61%), T3-4: 17 (39%)	N0-2a: 12 (26%), N2b-3: 34 (74%)	I-III: 20 (44%), IV: 36 (56%)	46 (100%)	0 (0%)	70 (58-70)	11 (24%)	46 (100%)	0 (0%)	N/A	N/A
Jensen et al. [15]	R	2010-2014	MDACC	114	N/A (N/A)	N/A (N/A)	N/A	N/A	63 (55%)	51 (45%)	N/A	98 (86%)	16 (14%)	85 (51%)	48 (49%)	T1-2: 86 (75%), T3-4: 28 (25%)	N0-1: 18 (16%), N2-3: 96 (84%)	N/A	114 (100%)	0 (%)	N/A (N/A)	54 (47%)	96 (84%)	N/A	N/A	N/A
Zhang et al. [16]	R	2011-2014	MDACC	534	33.8 (N/A)	N/A (N/A)	462 (87%)	72 (13%)	N/A	N/A	N/A	364 (68%)	170 (32%)	260 (49%)	274 (51%)	T1-2: 347 (65%), T3-4: 187 (35%)	N0-1: 92 (17%), N2-3: 442 (83%)	N/A	534 (100%)	0 (0%)	N/A (N/A)	217 (41%)	360 (67%)	N/A	N/A	N/A
Sharma et al. [17]	R	2013-2015	UPenn	33	N/A (N/A)	58 (N/A)	27 (82%)	6 (18%)	N/A	N/A	N/A	N/A	N/A	13 (39%)	20 (61%)	T1-2: 32 (97%), T3-4: 1 (3%)	N0-2b: 30 (91%), N2c-3: 3 (9%)	I-III: 5 (15%), IV: 28 (85%)	0 (0%)	33 (100%)	61.7 (N/A)	N/A	14 (42%)	19 (58%)	N/A	0 (0%)
Rwigema et al. [19]	R	2013-2016	UPenn	175	N/A (N/A)	60.1 (N/A)	112 (64%)	63 (36%)	N/A	N/A	N/A	N/A	N/A	N/A	N/A	T1-2: 85 (49%), T3-4: 90 (51%)	N0: 40 (23%), N1-3: 135 (77%)	N/A	175 (100%)	0 (0%)	69.8 (N/A)	N/A	101 (58%)	74 (42%)	N/A	N/A
Manzar et al. [22]	R	2013-2018	Mayo	259	30 (N/A)	61 (42-88)	224 (86%)	35 (14%)	93 (36%)	165 (63%)	1 (1%)	219 (85%)	40 (15%)	125 (48%)	134 (52%)	T1-2: 152 (59%), T3-4: 102 (39%)	N0-1: 37 (14%), N2-3: 222 (86%)	I-III: 26 (10%), IV: 232 (90%)	111 (43%)	148 (57%)	70 (56-70)	N/A	173 (67%)	86 (33%)	155 (60.3%)	N/A
Cao et al. [24]	R	2011-2015	MDACC	429	35.8 (N/A)	59 (32-84)	368 (86%)	61 (14%)	N/A	N/A	N/A	295 (69%)	134 (31%)	203 (47%)	223 (53%)	T1-2: 292 (68%), T3-4: 137 (32%)	N0-1: 76 (18%), N2-3: 353 (82%)	N/A	429 (100%)	0 (0%)	N/A (N/A)	158 (37%)	289 (67%)	N/A	N/A	N/A
Anderson et al. [5]	P	2016-2019	Mayo	17	N/A (N/A)	N/A (N/A)	N/A	N/A	N/A	N/A	N/A	16 (100%)	0 (0%)	N/A	N/A	T1-2: 16 (100%), T3-4: 0 (0%)	N/A	N/A	0 (0%)	16 (100%)	60 (54-63)	N/A	N/A	N/A	2 (11.8%)	N/A
Youssef et al. [26]	R	2018-2021	MSKCC	234	26 (N/A)	64.1 (N/A)	204 (87%)	31 (13%)	117 (50%)	117 (50%)	N/A	215 (92%)	19 (8%)	N/A	N/A	T1-2: 157 (67%), T3-4: 77 (33%)	N0-1: 53 (23%), N2-3: 181 (77%)	N/A	234 (100%)	0 (0%)	N/A (N/A)	N/A	228 (97%)	5 (3%)	29 (12.4%)	4 (1.6%)

**Table 3:** Quality of the included studies by MINORS criteria MINORS: Methodological Index for Non-randomized Studies; R: retrospective; P: prospective; N/A: not available

Author (citation)	Study type	Clearly stated aim	Inclusion of consecutive patients	Prospective data collection	Endpoints appropriate of study	Unbiased assessment of study endpoint	Follow-up period appropriate to study aim	Loss to follow-up does not exceed the proportion experiencing the major endpoint	An adequate control group	Contemporary groups	Baseline equivalence of groups	Adequate statistical analysis	Total score
Blanchard et al. [13]	R	2	2	N/A	2	1	2	2	2	2	2	2	19
Gunn et al. [4]	P	2	2	2	2	1	2	2	N/A	N/A	N/A	2	15
Sio et al. [14]	R	2	2	N/A	2	1	2	2	2	2	2	2	19
Jensen et al. [15]	R	2	2	N/A	2	2	2	2	2	2	2	2	20
Zhang et al. [16]	R	2	2	N/A	2	1	1	2	2	2	2	2	18
Sharma et al. [17]	R	2	2	N/A	2	1	2	2	2	2	2	2	19
Aljabab et al. [18]	R	2	2	N/A	2	1	2	2	N/A	N/A	N/A	2	13
Rwigema et al. [19]	R	2	2	N/A	2	1	2	2	2	1	1	2	17
Bagley et al. [20]	R	2	2	N/A	2	1	2	2	N/A	N/A	N/A	2	13
Grant et al. [21]	R	2	2	N/A	2	1	2	2	N/A	N/A	N/A	2	13
Manzar et al. [22]	R	2	2	N/A	2	1	2	2	2	2	2	2	19
Bahig et al. [23]	R	2	2	N/A	2	1	2	2	N/A	N/A	N/A	2	13
Cao et al. [24]	R	2	2	N/A	2	1	2	2	N/A	N/A	N/A	2	13
Wright et al. [25]	R	2	2	N/A	2	1	2	2	N/A	N/A	N/A	2	13
Anderson et al. [5]	P	2	2	2	2	1	2	2	2	2	0	2	19
Youssef et al. [26]	R	2	2	N/A	2	1	2	2	2	2	2	2	19
Pollock et al. [27]	R	2	2	N/A	2	1	2	2	N/A	N/A	N/A	2	13
Singh et al. [6]	R	2	2	N/A	2	1	2	2	N/A	N/A	N/A	2	13

Acute and Late Toxicities Following PRT

Toxicity outcomes were reported in 13 studies [4-6,13,15-19,21,22,26,27] consisting of 356 patients. The majority of patients were men (90%) with HPV-associated disease (93%) who received treatment in the definitive setting (68%). Per AJCC 7th Edition, 72% of patients had T1-2 disease and 71% had N2-3; the most common primary tumor site was tonsil (54%). Among all studies, toxicities were graded by clinicians using the Common Terminology Criteria for Adverse Events (CTCAE) Version 4 or 5.

**Figure 2:**
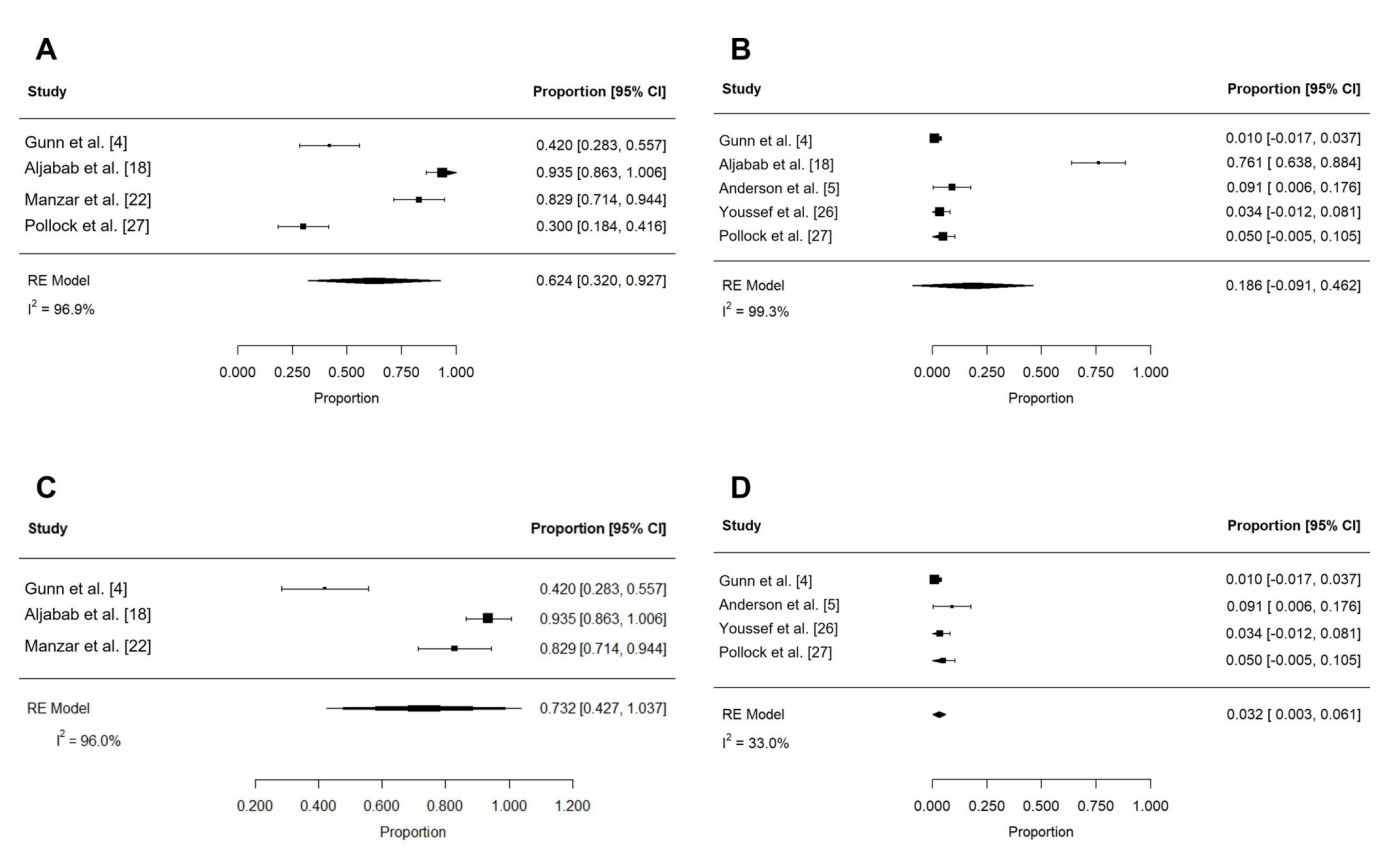
Pooled rates of acute dermatitis after PRT Pooled estimates of acute (A) grade 2 or more and (B) grade 3 or more dermatitis after PRT. Given heterogeneity (I2 > 50%), rates were re-estimated after the removal of outlier studies, which were identified using Cook's distance (C-D). PRT: proton radiotherapy

**Figure 3:**
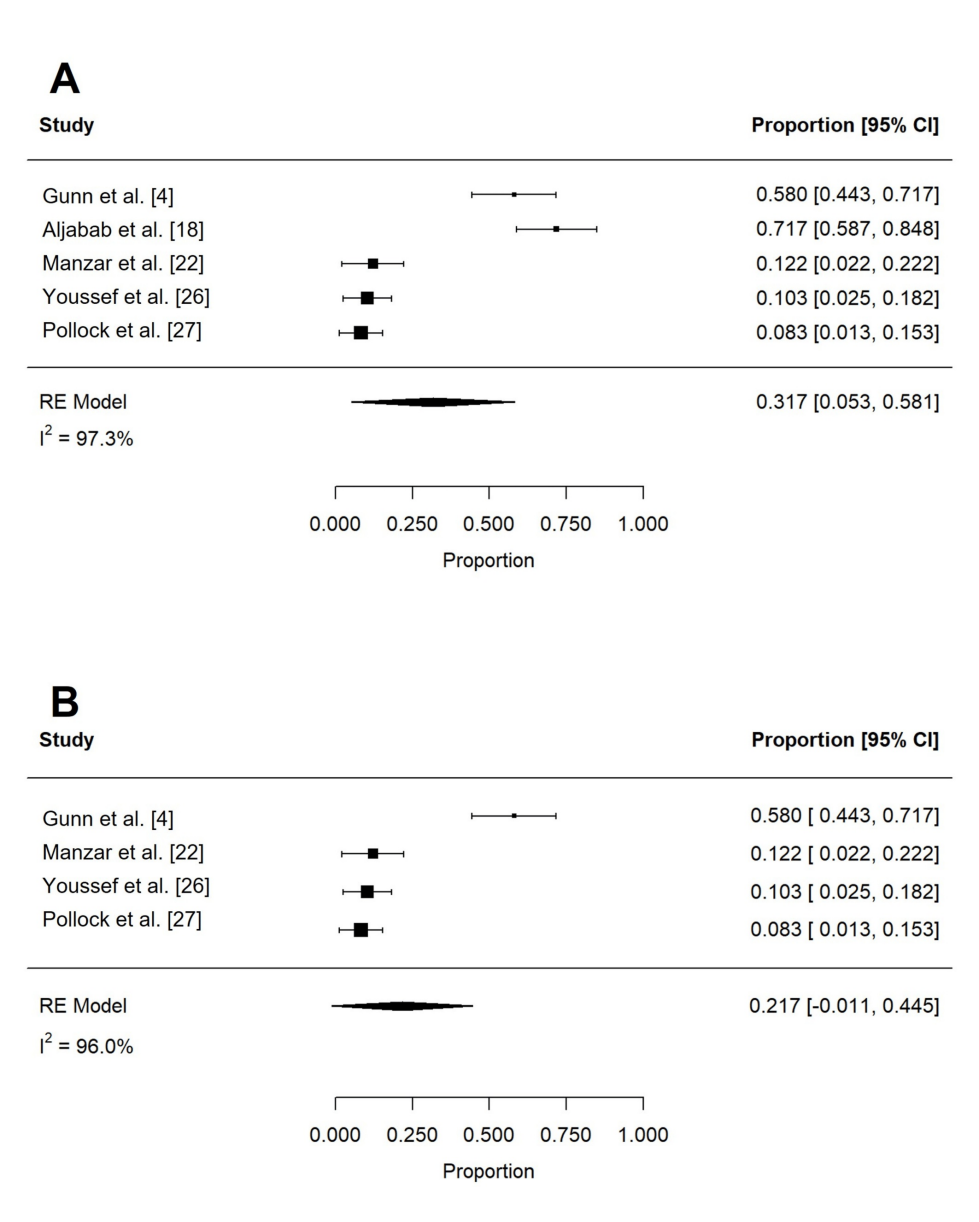
Pooled rates of acute mucositis after PRT Pooled estimates of acute (A) grade 3 or more mucositis after PRT. Given heterogeneity (I2 > 50%), rates were re-estimated after the removal of outlier studies, which were identified using Cook's distance (B). PRT: proton radiotherapy

**Figure 4:**
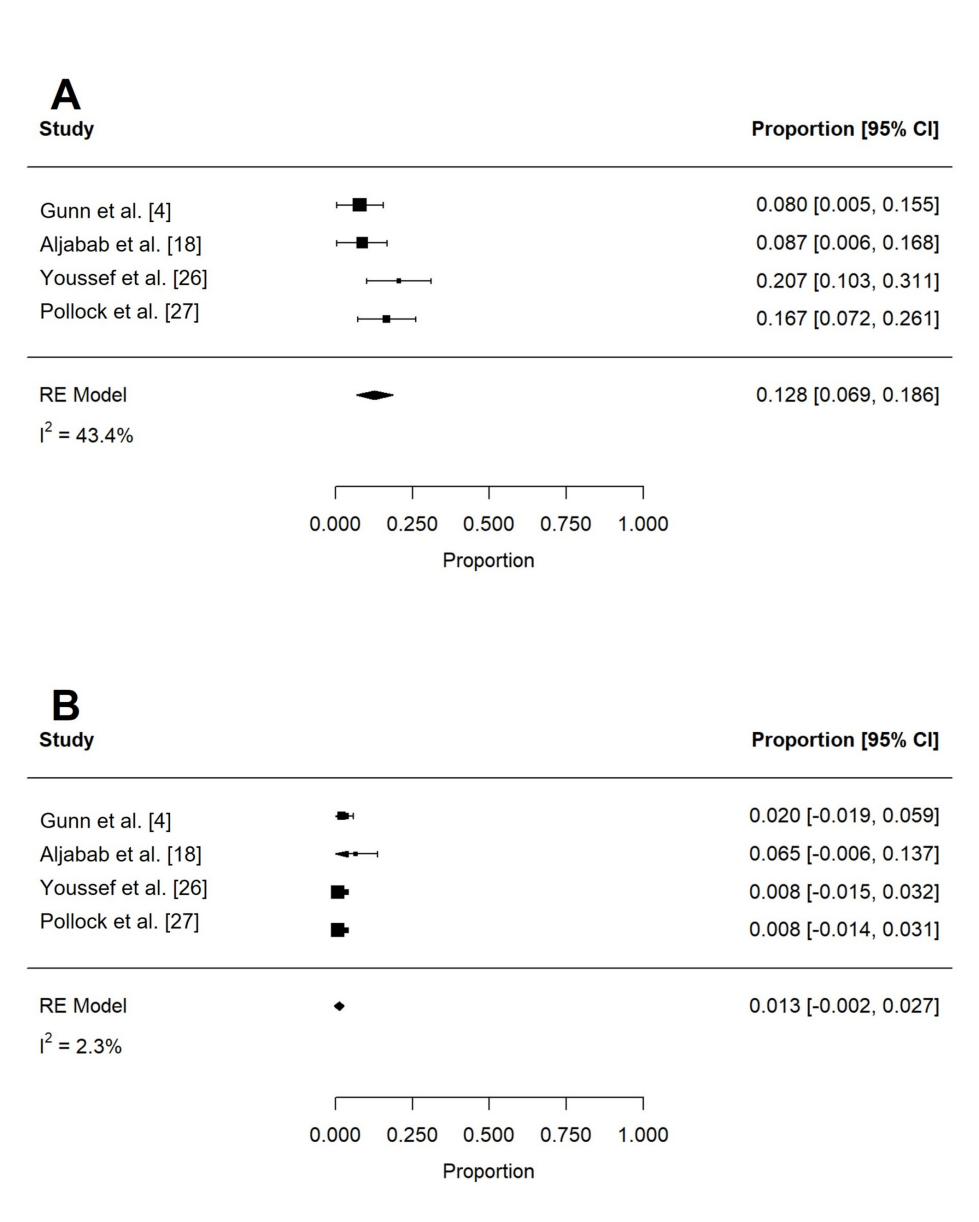
Pooled rates of acute xerostomia after PRT Pooled estimates of acute (A) grade 2 or more and (B) grade 3 or more xerostomia after PRT. PRT: proton radiotherapy

**Figure 5:**
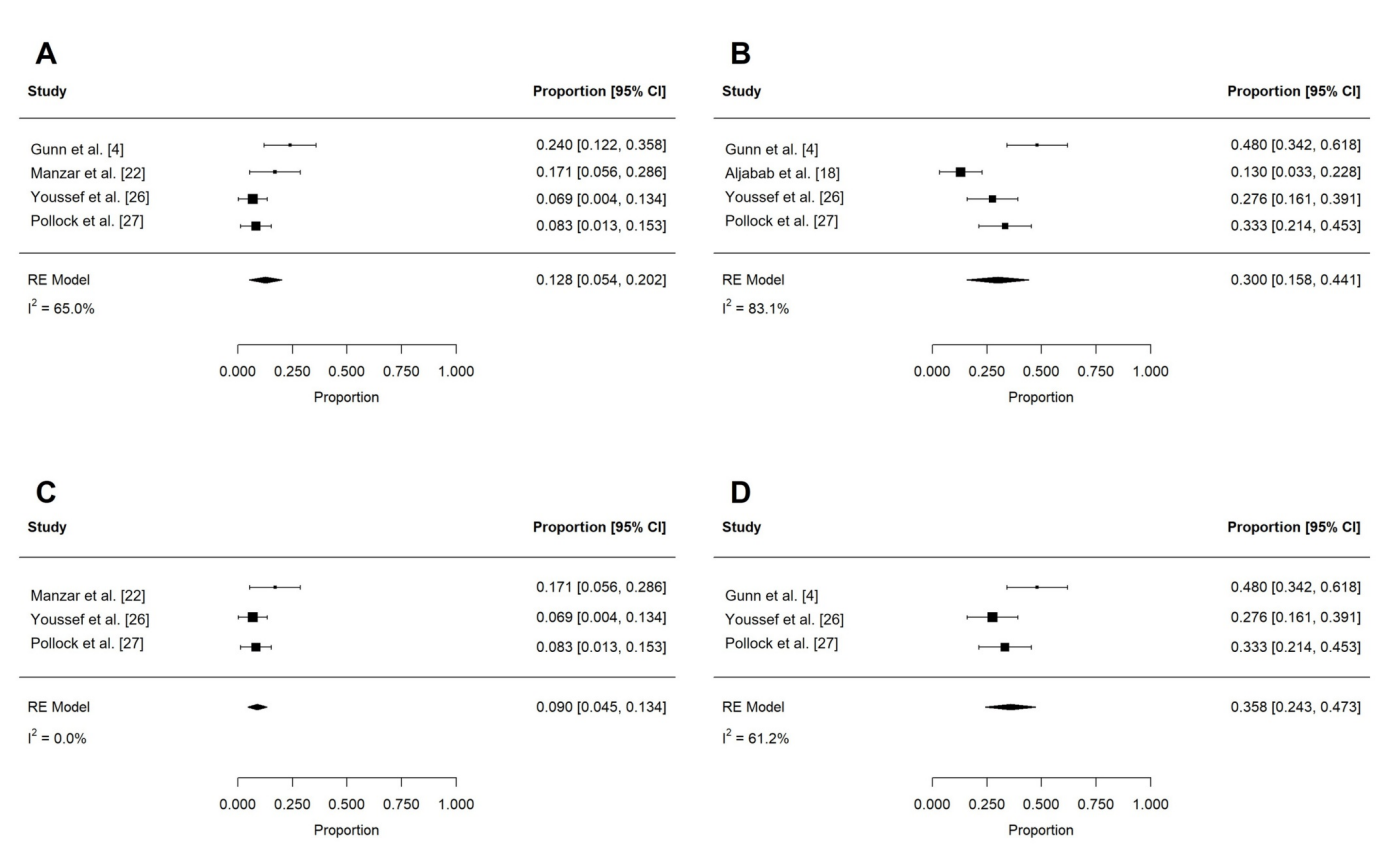
Pooled rates of acute dysphagia and dysgeusia after PRT Pooled estimates of acute (A) grade 3 or more dysphagia and (B) grade 2 or more dysgeusia after PRT. Given heterogeneity (I2 > 50%), rates were re-estimated after the removal of outlier studies, which were identified using Cook's distance (C-D). PRT: proton radiotherapy

**Figure 6:**
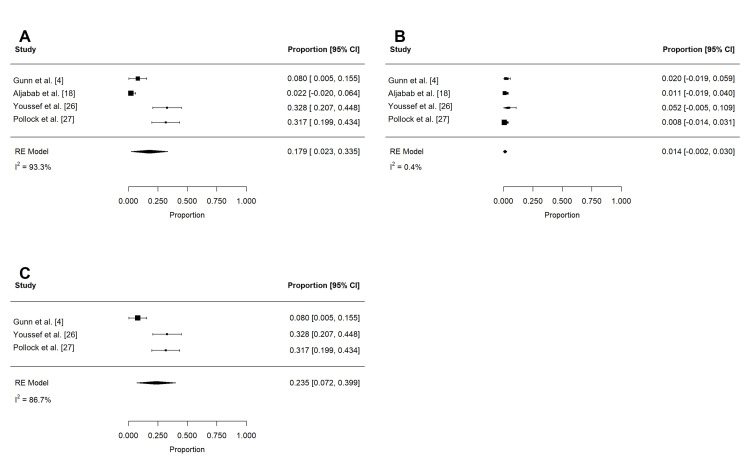
Pooled rates of acute weight loss after PRT Pooled estimates of acute (A) grade 2 and (B) grade 3 weight loss after PRT. Given heterogeneity (I2 > 50%), rates of (C) grade 2 weight loss were re-estimated after the removal of outlier studies, which were identified using Cook's distance. PRT: proton radiotherapy

**Figure 7:**
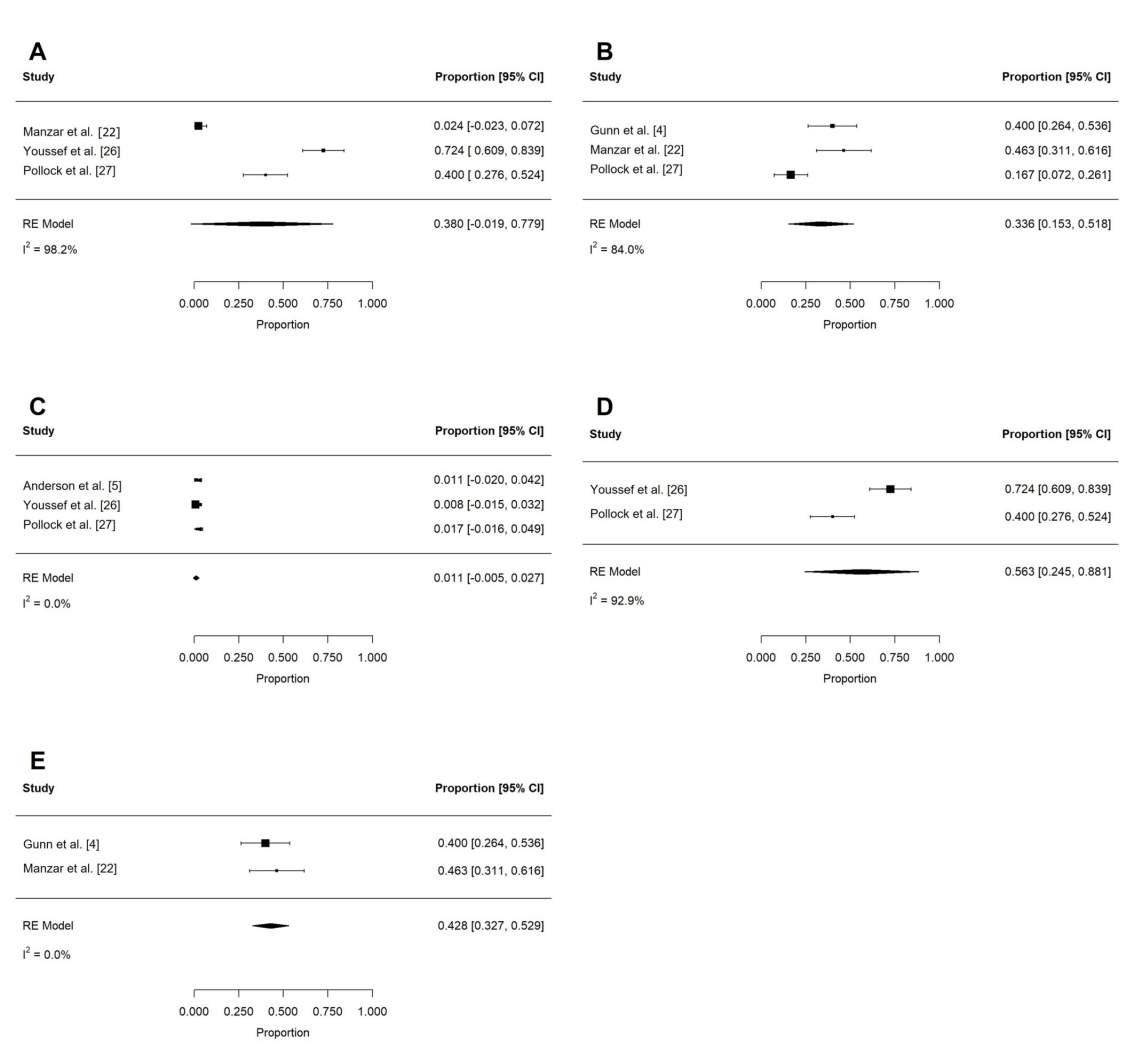
Pooled rates of acute pain, fatigue, and nausea after PRT Pooled estimates of late (A) grade 2 or more pain, (B) grade 2 or more fatigue, and (C) grade 3 nausea after PRT. Given heterogeneity (I2 > 50%), rates of (D) grade 2 or more pain and (E) grade 2 or more fatigue were re-estimated after the removal of outlier studies, which were identified using Cook's distance. PRT: proton radiotherapy

**Figure 8:**
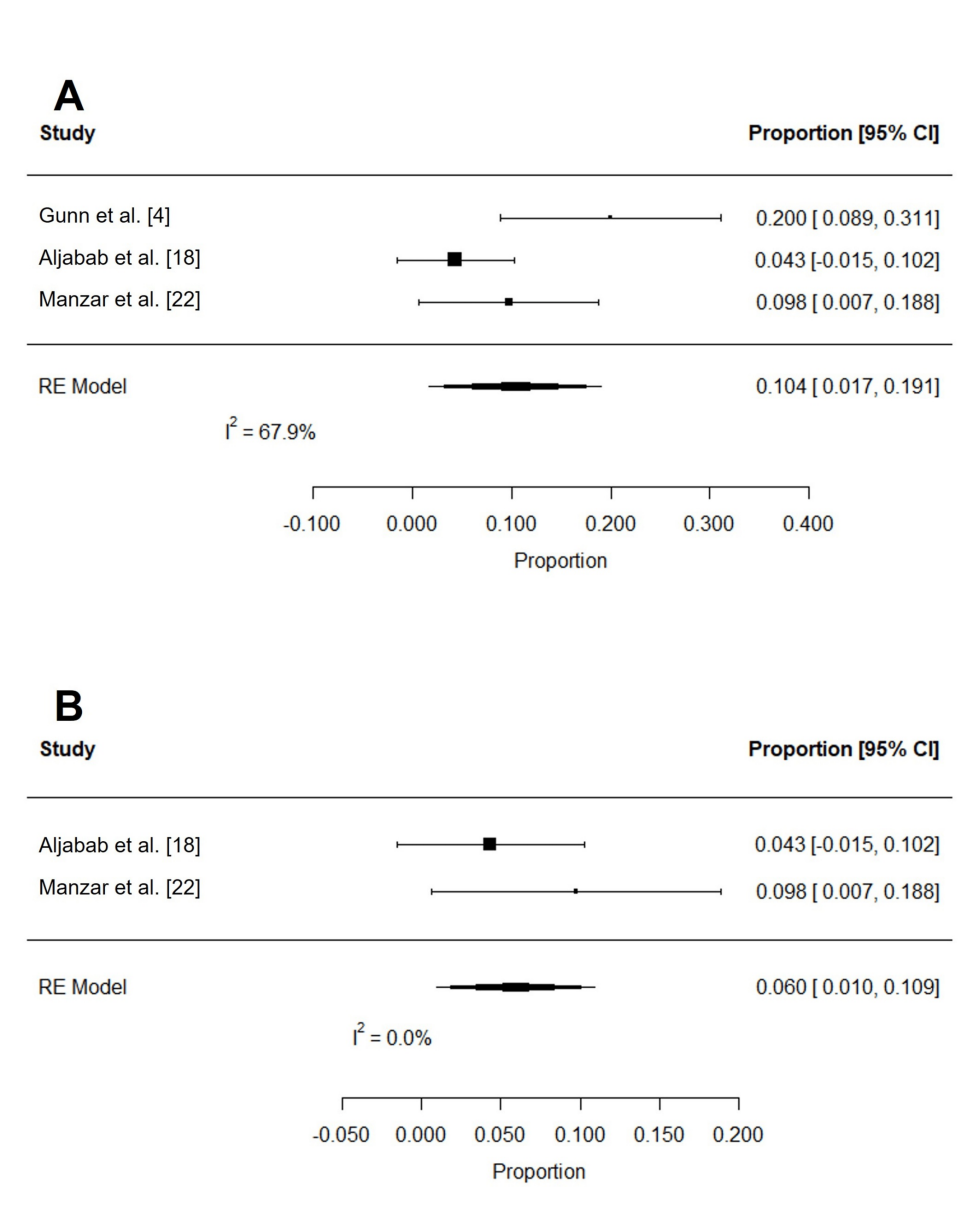
Pooled rates of acute hospitalization after PRT Pooled estimates of (A) acute hospitalization (within 30 days) after PRT. Given heterogeneity (I2 > 50%), rates were re-estimated after the removal of outlier studies, which were identified using Cook's distance (B). PRT: proton radiotherapy

Acute toxicities reported in at least three studies are summarized in Table 4 and Table 5. These included dermatitis, mucositis, xerostomia, dysphagia, weight loss, dysgeusia, nausea, pain, fatigue, and feeding tube use. To reduce confounding, if studies contained overlapping patient populations, only the larger study was included for statistical analysis. Rates of grade 2 or more (n = 197) and grade 3 or more (n = 258) dermatitis were 62.4% (95% confidence interval (CI): 32.0 to 92.7%; I2 = 96.9%) and 18.6% (95% CI: -9.1 to 46.2%; I2 = 99.3%), respectively (Figure 2). The rate of grade 3 or more mucositis (n = 255) was 31.7% (95% CI: 5.3 to 58.1%; I2 = 97.3%; Figure 3). Acute xerostomia of grade 2 or more (n = 214) and grade 3 or more (n = 214) were estimated to be 12.8% (95% CI: 6.9 to 18.6%; I2 = 43.4%) and 1.3% (95% CI: -0.2 to 2.3%; I2 = 2.3%), respectively (Figure 4). The pooled rate of grade 3 or more dysphagia (n = 209) was 12.8% (95% CI: 5.4 to 20.2%; I2 = 65%; Figure 5A), while the rate of grade 2 dysgeusia (n = 214) was 30.0% (95% CI: 15.8 to 44.1%; I2 = 83.1%; Figure 5B). Weight loss was grade 2 (n = 214) and grade 3 (n = 214) in 17.9% (95% CI: 2.3 to 33.5%; I2 = 93.3%) and 1.4% (95% CI: -0.2 to 3.0%; I2 = 0.4%), respectively (Figure 6). Acute pain (grade 2 or more; n = 159), fatigue (grade 2 or more; n = 151), and nausea (grade 3; n = 162) were 38.0% (95% CI: -1.9 to 77.9%; I2 = 98.2%; Figure 7A), 33.6% (95% CI: 15.3 to 51.8%; I2 = 84%; Figure 7B), and 1.1% (95% CI: -0.5 to 2.7%; I2 = 0.0%; Figure 7C), respectively. The pooled rate of hospitalization during or within 30 days of PRT (n = 137) was 10.4% (95% CI: 1.7 to 19.1%; I2 = 67.9%; Figure 8).

**Table 4:** Pooled rates of acute toxicities associated with PRT PRT: proton radiotherapy; CTCAE: Common Terminology Criteria for Adverse Events; N/A: not applicable

Adverse event	CTCAE grade	Total patients	Pooled rate (95% confidence interval)
Dermatitis	2+	197	62.4% (32.0 to 92.7%)
	3+	258	18.6% (-12.8 to 56.9%)
Mucositis	3+	255	31.7% (5.3 to 58.1%)
Xerostomia	2+	214	12.8% (6.9 to 18.6%)
	3+	214	1.3% (-0.2 to 2.3%)
Dysphagia	3+	209	12.8% (5.4 to 20.2%)
Dysgeusia	2	214	30.0% (15.8 to 44.1%)
Weight loss	2	214	17.9% (2.3 to 33.5%)
	3	214	1.4% (-0.2 to 3.0%)
Pain	2+	159	38.0% (-1.9 to 77.9%)
Fatigue	2+	151	33.6% (15.3 to 51.8%)
Nausea	3	162	1.1% (-0.5 to 2.7%)
Hospitalization (within 30 days)	N/A	137	10.4% (1.7 to 19.1%)
Feeding tube use		239	21.4% (5.3 to 37.6%)

**Table 5:** Acute toxicities reported in at least three studies CTCAE: Common Terminology Criteria for Adverse Events; N/A: not available

Author (citation)	Toxicity scale	Total	Dermatitis		Mucositis	Xerostomia		Dysphagia	Pain	Fatigue	Nausea	Weight loss		Dysgeusia	Hospitalization (within 30 days)
			Grade 2+	Grade 3+	Grade 3+	Grade 2+	Grade 3+	Grade 3+	Grade 2+	Grade 2+	Grade 3+	Grade 2	Grade 3	Grade 2	
Gunn et al. [4]	CTCAE v.4	50	21 (42%)	0 (0%)	29 (58%)	4 (8%)	1 (2%)	12 (24%)	N/A	20 (40%)	N/A	4 (8%)	1 (2%)	24 (48%)	10 (20%)
Aljabab et al. [18]	CTCAE v.4	46	43 (93%)	35 (75%)	33 (71%)	4 (9%)	3 (7%)	N/A	N/A	N/A	N/A	1 (2%)	0 (0%)	6 (13%)	2 (4%)
Manzar et al. [22]	CTCAE v.4	41	34 (83%)	N/A	5 (12%)	N/A	N/A	7 (17%)	1 (2%)	19 (46%)	N/A	N/A	N/A	N/A	4 (9%)
Anderson et al. [5]	CTCAE v.4	44	N/A	4 (9%)	N/A	N/A	N/A	N/A	N/A	N/A	0 (0%)	N/A	N/A	N/A	N/A
Youssef et al. [26]	CTCAE v.4-5	58	N/A	2 (3%)	6 (10%)	12 (20%)	0 (0%)	4 (7%)	42 (72%)	N/A	0 (0%)	19 (32%)	3 (5%)	16 (28%)	N/A
Pollock et al. [27]	CTCAE v. 5	60	18 (30%)	3 (5%)	5 (8%)	10 (17%)	0 (0%)	5 (8%)	24 (40%)	10 (17%)	1 (2%)	19 (32%)	0 (0%)	20 (33%)	N/A

**Figure 9:**
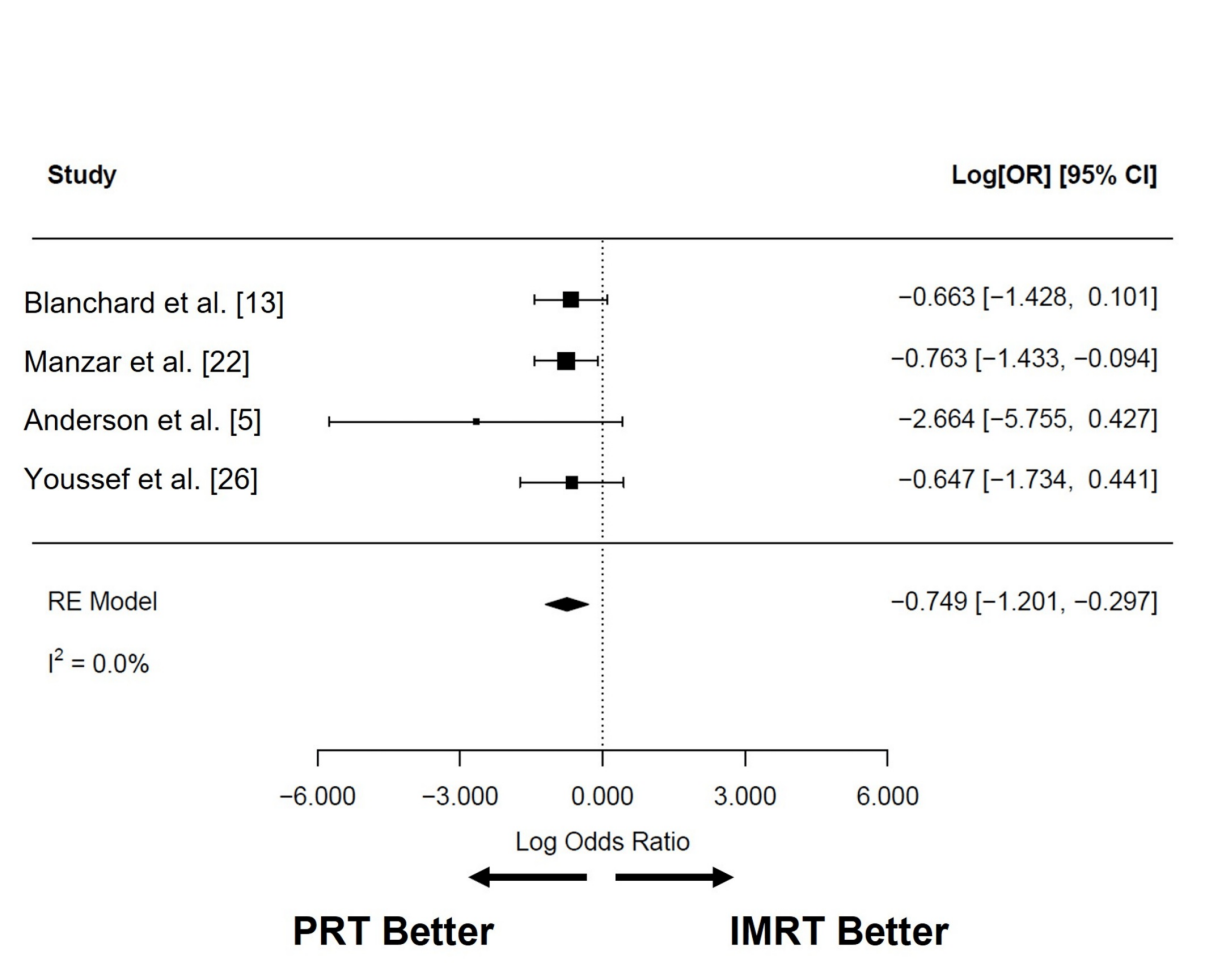
Comparison of acute feeding tube use after PRT and IMRT Ratios less than 0 indicate lower odds of feeding tube use in patients treated with PRT. PRT: proton radiotherapy; IMRT: intensity-modulated radiotherapy

Among all the reported toxicities, feeding tube use was the only acute toxicity reported in at least three studies that included patients treated with PRT or IMRT. Following IMRT (n = 608), the pooled rate of feeding tube use was 31.0% (95% CI: 8.0 to 54.0%; I2 = 97.5%), while after PRT (n = 239), it was 21.4% (95% CI: 5.3 to 37.6%; I2 = 95.0%). Compared to IMRT, PRT was associated with a significantly lower feeding tube use (log OR: -0.749; 95% CI: -1.201 to -0.297; I2 = 0%; P = 0.0012; Figure 9), corresponding to a 2.12-fold lower odds of feeding tube use in the acute setting (absolute difference 10%).

**Figure 10:**
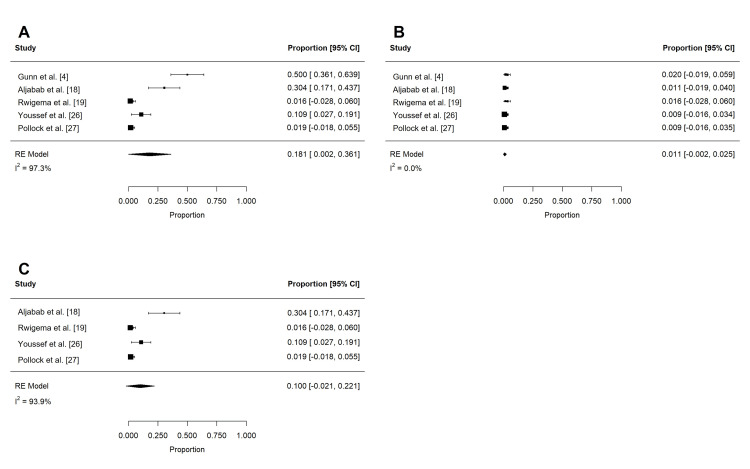
Pooled rates of late xerostomia after PRT Pooled estimates of late (A) grade 2 and (B) grade 3 xerostomia after PRT. Given heterogeneity (I2 > 50%), rates of grade 2 xerostomia were re-estimated after the removal of outlier studies, which were identified using Cook's distance (C). PRT: proton radiotherapy

**Figure 11:**
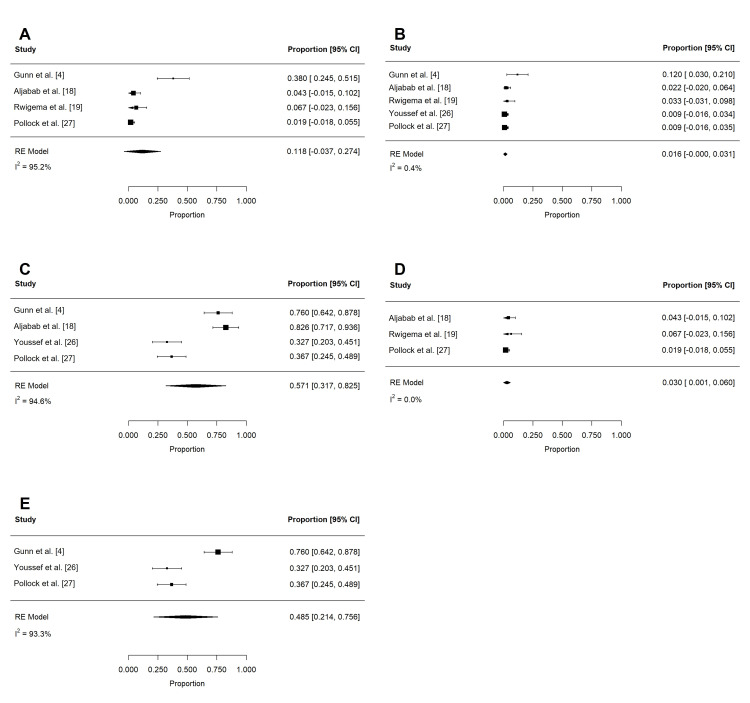
Pooled rates of late dysphagia and dysgeusia after PRT Pooled estimates of late (A) grade 2 or more dysphagia, (B) grade 3 or more dysphagia, and (C) grade 1-2 dysgeusia after PRT. Given heterogeneity (I2 > 50%), rates of (D) grade 2 or more dysphagia and (E) grade 1-2 dysgeusia were re-estimated after the removal of outlier studies, which were identified using Cook's distance. PRT: proton radiotherapy

**Figure 12:**
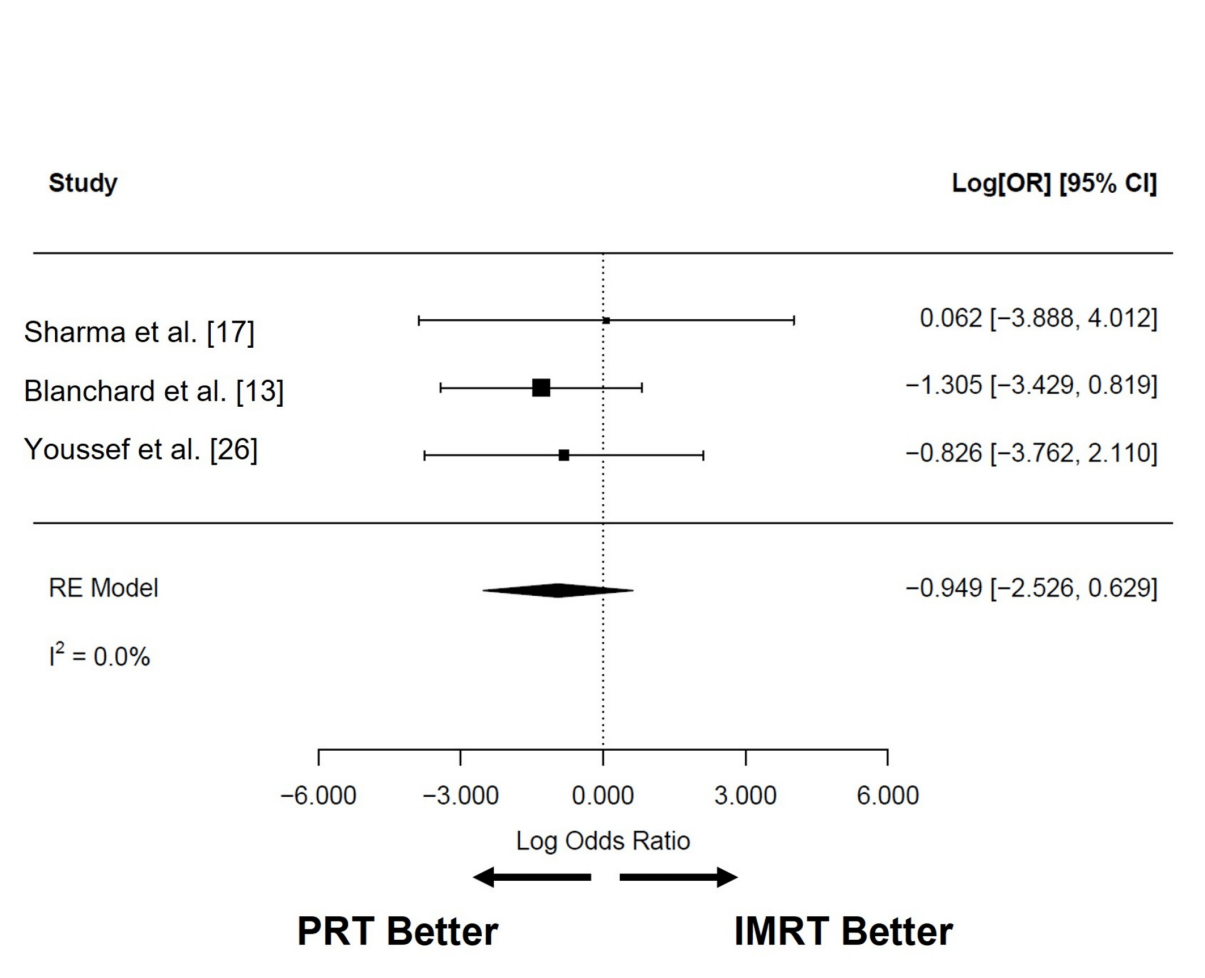
Comparison of late feeding tube use after PRT and IMRT Ratios less than 0 indicate lower odds of feeding tube use in patients treated with PRT. PRT: proton radiotherapy; IMRT: intensity-modulated radiotherapy

Late toxicities after PRT reported in at least three studies were xerostomia, dysphagia, dysgeusia, and feeding tube use (Table 6 and Table 7). Grade 2 (n = 234) and 3 (n = 234) xerostomia were estimated to be 18.1% (95% CI: 0.2 to 36.1%; I2 = 97.3%) and 1.1% (95% CI: -0.2 to 2.5%; I2 = 0%), respectively (Figure 10). Dysphagia of grades 2 or more (n = 179) and 3 or more (n = 234) were 11.8% (95% CI: -3.7 to 27.4%; I2 = 95.2%) and 1.6% (95% CI: 0.0 to 3.1%; I2 = 0.4%), respectively (Figure 11A-11B). The pooled rate of late grade 1-2 dysgeusia (n = 211) was 57.1% (95% CI: 31.7 to 82.5%; I2 = 94.6%; Figure 11C). Osteoradionecrosis (ORN) was reported among four studies [4,6,16,26]: while crude rates of ORN ranged from 0% to 9.4%, the pooled analysis could not be completed due to overlapping patient populations. Among late toxicities, only feeding tube use was reported in at least three studies that included patients treated with PRT or IMRT. At six months or more after RT, the pooled rates of late feeding tube use associated with PRT (n = 185; 1.4% (95% CI: -0.3 to 3.0; I2 = 0%)) and IMRT (n = 367; 2.7% (95% CI: 0 to 5.4%; I2 = 48.5%)) were not significantly different (log OR: -0.949 (95% CI: -2.526 to 0.629); I2 = 0%; P = 0.239; Figure 12).

**Table 6:** Pooled rates of late toxicities associated with PRT PRT: proton radiotherapy; CTCAE: Common Terminology Criteria for Adverse Events; N/A: not applicable

Adverse event	CTCAE grade	Total patients	Pooled rate (95% confidence interval)
Xerostomia	2	234	18.1% (0.2 to 36.1%)
	3	234	1.1% (-0.2 to 2.5%)
Dysphagia	2+	179	11.8% (-3.7 to 27.4%)
	3+	234	1.6% (0.0 to 3.1%)
Dysgeusia	1-2	211	57.1% (31.7 to 82.5%)
Feeding tube use	N/A	185	1.4% (-0.3 to 3.0%)

**Table 7:** Late toxicities reported in at least three studies *, **: overlapping patient populations. CTCAE: Common Terminology Criteria for Adverse Events; N/A: not available

Author (citation)	Toxicity scale	Total	Xerostomia		Dysphagia		Dysgeusia	Osteoradionecrosis
			Grade 2	Grade 3	Grade 2+	Grade 3+	Grade 1-2	Grade 1+
Gunn et al. [4]*	CTCAE v.4	50	25 (50%)	1 (2%)	19 (38%)	6 (12%)	38 (76%)	1 (2%)
Zhang et al. [16]*	CTCAE v.4	50	N/A	N/A	N/A	N/A	N/A	1 (2%)
Aljabab et al. [18]	CTCAE v.4	46	14 (30%)	0 (0%)	2 (4.3%)	1 (2.2%)	38 (83%)	N/A
Rwigema et al. [19]	CTCAE v.4	30	0 (0%)	0 (0%)	2 (6.7%)	1 (3.3%)	N/A	N/A
Youssef et al. [26]**	CTCAE v.4-5	55	6 (11%)	0 (0%)	N/A	0 (0%)	18 (33%)	1 (2%)
Pollock et al. [27]	CTCAE v.5	53	1 (2%)	0 (0%)	1 (2%)	0 (0%)	22 (42%)	N/A
Singh et al. [6]**	Glanzmann and Gratz	53	N/A	N/A	N/A	N/A	N/A	5 (9.4%)

Oncologic Outcomes Associated With PRT

**Figure 13:**
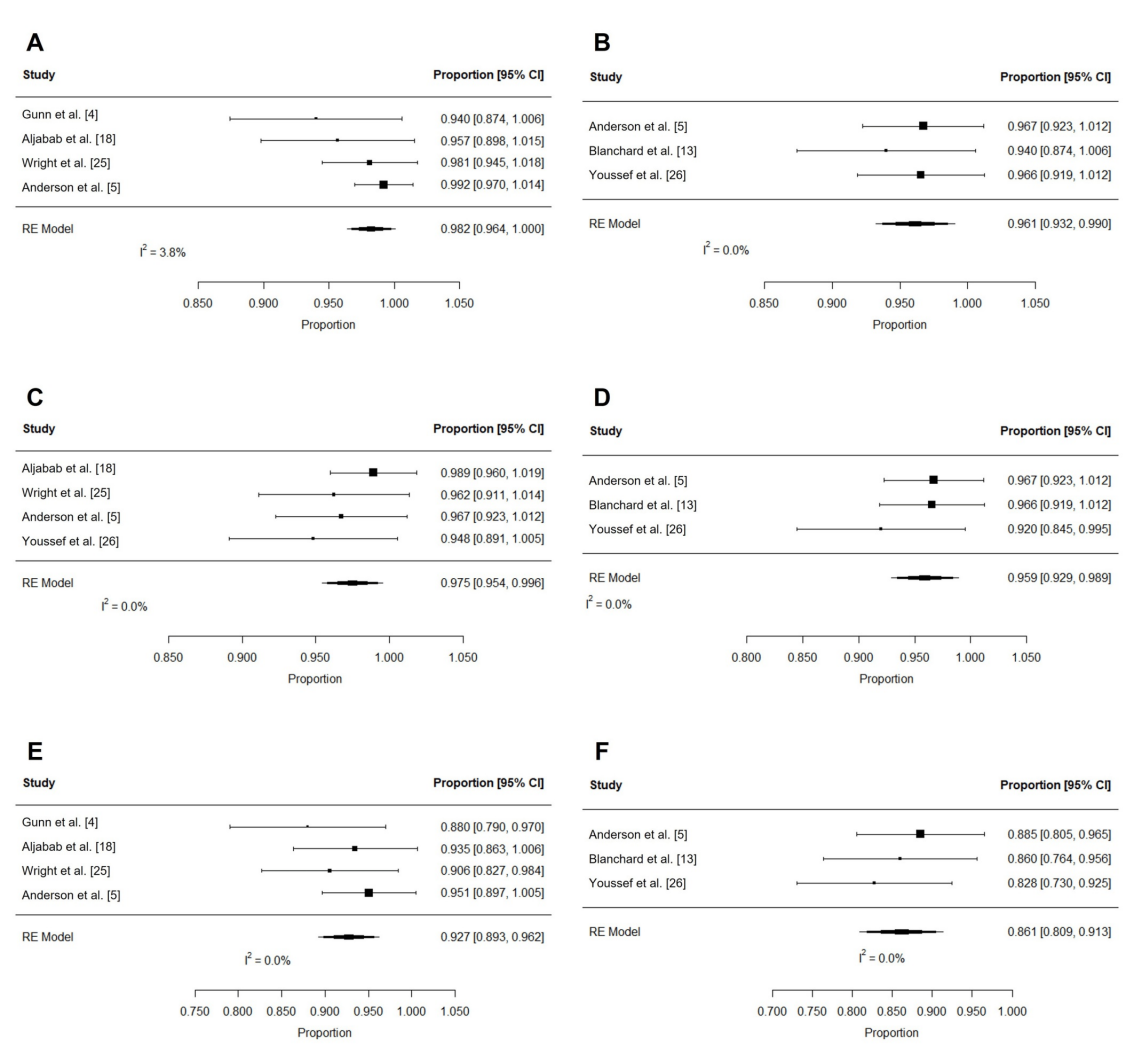
Pooled oncologic outcomes after PRT Pooled estimates of actuarial two- and three-year (A-B) overall survival, (C-D) locoregional control, and (E-F) progression-free survival after PRT. PRT: proton radiotherapy

Table 8 and Table 9 summarize the oncologic outcomes and patterns of failure associated with PRT. Actuarial outcomes were reported in eight studies (314 patients) [4,5,13,15,18,22,25,26]. Following PRT, estimated OS at two years (n = 210) and three years (n = 169) was high (Figure 13A-13B): 98.2% (95% CI: 96.4 to 100%; I2 = 3.8%) and 96.1% (95% CI: 93.2 to 99%; I2 = 0%), respectively. Similarly, locoregional control at two years (n = 218) was 97.5% (95% CI: 95.4 to 99.6%; I2 = 0%) and at three years (n = 169) was 95.9% (95% CI: 92.9 to 98.9%; I2 = 0%; Figure 13C-13D). After proton beam therapy (PBT), pooled PFS at two years (n = 210) was 92.7% (95% CI: 89.3 to 96.2%; I2 = 0%) and at three years (n = 169) was 86.1% (95% CI: 80.9 to 91.3%; I2 = 0%; Figure 13E-13F).

**Table 8:** Oncologic outcomes associated with PRT PRT: proton radiotherapy; N/A: not applicable

Survival outcome	Time post-PRT	Total patients	Pooled estimate (95% confidence interval)
Overall survival	2 years	210	98.2% (96.4 to 100%)
	3 years	169	96.1% (93.2 to 99%)
Locoregional control	2 years	218	97.5% (95.4 to 99.6%)
	3 years	169	95.9% (92.9 to 98.9%)
Progression-free survival	2 years	210	92.7% (89.3 to 96.2%)
	3 years	169	86.1% (80.9 to 91.3%)
Crude locoregional failures	N/A	276	2.1% (0.4 to 3.8%)
Crude distant failures		233	5.9% (2.8 to 9.0%)

**Table 9:** Oncologic outcomes reported in at least three studies N/A: not available

Author (citation)	Total	Overall survival		Progression-free survival		Locoregional control		Crude failures	
		2 years	3 years	2 years	3 years	2 years	3 years	Locoregional	Distant
Gunn et al. [4]	50	94.5%	N/A	88.6%	N/A	N/A	N/A	N/A	N/A
Blanchard et al. [13]	50	N/A	94.3%	N/A	86.4%	N/A	91%	N/A	N/A
Aljabab et al. [18]	46	95.7%	N/A	93.5%	N/A	100%	N/A	0 (0%)	2 (4.3%)
Bahig et al. [23]	56	N/A	N/A	N/A	N/A	N/A	N/A	5 (8.9%)	4 (7.1%)
Wright et al. [25]	53	98%	N/A	90.3%	N/A	97%	N/A	1 (1.9%)	N/A
Anderson et al. [5]	61	100%	97%	95%	88%	N/A	97%	1 (1.6%)	3 (4.9%)
Youssef et al. [26]	58	N/A	97%	N/A	82%	95%	96%	N/A	N/A
Pollock et al. [27]	60	N/A	N/A	N/A	N/A	N/A	N/A	1 (1.6%)	5 (8.3%)

Crude patterns of failure were reported in five studies (276 patients) [5,18,23,25,27]. Distant failures (n = 223) were most frequent (5.9%; 95% CI: 2.8 to 9.0%; I2 = 0.0%), followed by locoregional failures (n = 276 (2.1%); 95% CI: 0.4 to 3.8; I2 = 0.3%).

PROs Following PRT

PRO analysis was performed in eight studies [5,14,17,20-24] consisting of 456 and 784 patients treated with PRT and IMRT, respectively (Table 10). PROs were measured using eight instruments [28-34] and collected across multiple timepoints (Table 10). Only the QLQ-HN35 [30] instrument was reported in more than one study [5,17,22]. However, a pooled quantitative analysis could not be performed because data were collected at different timepoints after RT.

**Table 10:** Characteristics of the studies with PRO The timing of PRO collection is indicated by an x, representing PRO was collected at the given timepoint, or -, representing PRO was not collected at the given timepoint. MDACC: MD Anderson Cancer Center; PRT: proton radiotherapy; IMRT: intensity-modulated radiotherapy; PRO: patient-reported outcome; MDASI-HN: MD Anderson Symptom Inventory for Head and Neck Cancer; EORTC QLQ-HN35: European Organization for Research and Treatment of Cancer Quality of Life Questionnaire-Head and Neck 35; GRIX: Groningen Radiotherapy-Induced Xerostomia scale; XeQoLS: Xerostomia Quality of Life Scale; MDADI: MD Anderson Dysphagia Inventory; FACT-HN: Functional Assessment of Cancer Therapy-Head and Neck; XQ: Xerostomia Questionnaire; PROMIS10: Patient-Reported Outcomes Measurement Information System Global Health; N/A: not available; SD: standard deviation; IQR: interquartile range; UPenn: University of Pennsylvania

Author (citation)	Institution	No. of patients	PRT-IMRT comparison	PRO scale	Domains with data	Quantitative data	Timing of PRO collection (months)									
							Baseline	During RT	End of RT	2.5-3	6	9	12	18	24	36
Sio et al. [14]	MDACC	PRT: 35; IMRT: 46	Yes	MDASI-HN	Food taste, dry mouth, swallowing/chewing, fatigue, pain, appetite, mucus, sleep, mouth sores, drowsiness, distress	Mean (SD)	x	x	-	x	x					-
Sharma et al. [17]	UPenn	PRT: 31; IMRT: 33	Yes	EORTC QLQ-HN35, GRIX	EORTC: general health domain, physical and role function, overall xerostomia, dental issues, head and neck pain, fatigue, sticky saliva; GRIX: day/night xerostomia, sticky saliva day	Mean	x	-	-	x	x	-	x	-	-	-
Bagley et al. [20]	MDACC	PRT: 69; IMRT: N/A	No	XeQoLS	Global, physical, personal, pain, social	Mean (SD), IQR	x	x	-	x	x	-	x	-	x	-
Grant et al. [21]	MDACC	PRT: 71; IMRT: N/A	No	MDADI	Global, composite, emotional, functional, physical	Mean (SD), median	x	x	-	x	x	-	x	-	x	-
Manzar et al. [22]	Mayo	PRT: 46; IMRT: 259	Yes	EORTC QLQ-HN35	Cough, sense of smell/taste, problems with teeth, dry mouth, nutritional supplements, sexual symptoms, feeling ill, feeding tube, swallowing, pain, dry mouth, sticky saliva, weight loss	Mean difference	x	-	x	-	-	-	-	-	-	-
Bahig et al. [23]	MDACC	PRT: 57; IMRT: N/A	No	FACT-HN	General, total, trial outcome index, physical well-being, social/family well-being, emotional well-being, functional well-being, head and neck cancer concerns	Mean (SD)	x	x	-	x	x	-	x	-	x	-
Cao et al. [24]	MDACC	PRT: 103; IMRT: 429	Yes	XQ	N/A	Mean (SD), % XQ ≥ 50	-	-	-	x	x	x	x	x	x	x
Anderson et al. [5]	Mayo	PRT: 44; IMRT: 17	No	EORTC QLQ-HN35, PROMIS10	EORTC: pain, swallowing, problems with senses, speech problems, trouble with social eating, trouble with social contact, less sexuality, teeth, opening mouth, dry mouth, sticky saliva, coughing, felt ill, pain killers, nutritional supplements, feeding tube, weight loss, weight gain; PROMIS10: overall, physical, mental	Mean (SD), range	x	-	-	x	-	-	x	-	-	x

Qualitatively, there were notable trends in PROs with PRT. Among the three studies [20,21,23] that reported PRO changes over time, symptom severity was worst at week 6 during PRT, while changes plateaued between 12 and 24 months post-PRT. Comparisons in PROs between patients treated with either PRT or IMRT were reported in four studies [14,17,22,24], each using a different instrument assessment timepoint (Table 10). Compared to IMRT, these studies found that PRT was associated with improvement in multiple symptoms: xerostomia, dry mouth, taste, appetite, dental problems, sense of smell, use of nutritional supplements, and physical/role function (Table 11). However, most symptoms were improved at only one timepoint post-RT. The symptoms associated with improved PROs in multiple studies and timepoints were xerostomia and dry mouth (Table 11).

**Table 11:** Comparison of PRO between PRT and IMRT Only studies that directly compared PRT and IMRT are listed. For each instrument, comparisons are between baseline and the listed timepoint. Dash (-) indicated a comparison was not reported. MDACC: MD Anderson Cancer Center; PRT: proton radiotherapy; IMRT: intensity-modulated radiotherapy; PRO: patient-reported outcome; MDASI-HN: MD Anderson Symptom Inventory for Head and Neck Cancer; EORTC QLQ-HN35: European Organization for Research and Treatment of Cancer Quality of Life Questionnaire-Head and Neck 35; GRIX: Groningen Radiotherapy-Induced Xerostomia scale; XQ: Xerostomia Questionnaire; UPenn: University of Pennsylvania

Author (citation)	Institution	Total patients	PRO instrument	PROs								
				During RT	End of RT	2.5-3 months	6 months	9 months	12 months	18 months	24 months	36 months
Sio et al. [14]	MDACC	PRT: 35; IMRT: 46	MDASI-HN	No difference in the composite of subdomains between IMRT and PRT	-	PRT improved the composite of top 5 subdomains; PRT with improved food taste and appetite subdomains	No difference in the composite of subdomains between IMRT and PRT; PRT improved appetite subdomain					-
Sharma et al. [17]	UPenn	PRT: 31; IMRT: 33	EORTC QLQ-HN35, GRIX	-	-	PRT improved dental problems subdomain	PRT improved moderate-severe dry mouth, xerostomia day, xerostomia night, sticky saliva day, dental problems, physical function, role function subdomains	-	PRT improved pain, moderate-severe dry mouth, xerostomia day, role function subdomains	-	-	-
Manzar et al. [22]	Mayo	PRT: 46; IMRT: 259	EORTC QLQ-HN35	-	PRT improved cough, sense of smell/taste, nutritional supplements subdomains	-	-	-	-	-	-	-
Cao et al. [24]	MDACC	PRT: 103; IMRT: 429	XQ	-	-	No difference in xerostomia between IMRT and PRT				PRT improved xerostomia		

Discussion

PRT is an attractive treatment modality for patients with OPCs. Given that the majority of OPCs are HPV-related and long-term survival is high in these patients [28], the reduced doses to surrounding normal tissues afforded by PRT [1,3] could translate into lower toxicity rates and improved quality of life. PRT, however, is not without costs. It is an advanced RT modality that requires specialized equipment, facilities, personnel, and experience [29]. While the number of proton facilities is increasing [30,31], travel to these centers poses a barrier to access [32]. Moreover, the cost-effectiveness of PRT compared to IMRT is widely debated [9,33,34]. While awaiting final results from randomized controlled trials comparing PRT to IMRT [8,35,36], additional data are needed to inform patient and provider decisions regarding the use of PRT in the upfront management of OPCs. 

In this study, oncologic outcomes associated with PRT appear to be similar to those reported in prospective studies of IMRT (Table 12). For example, in RTOG-1016, IMRT with concurrent cisplatin for patients with locally advanced HPV-associated OPC resulted in five-year OS and PFS rates of 84.6% and 78.4%, respectively [37]. In NRG-HN002, patients with low-risk, early-stage HPV-associated OPCs who received de-escalated IMRT with or without concurrent cisplatin had two-year OS rates of 96.7% and 97.3%, respectively, and two-year PFS rates of 90.5% and 87.6%, respectively [38]. While RTOG-1016 and NRG-HN002 used different RT doses (70 and 60 Gy, respectively), these were comparable to doses used by studies included in this analysis (Table 1 and Table 2). Moreover, for patients treated in these trials with IMRT and concurrent cisplatin, distant and locoregional failure patterns were similar to those observed in our study (Table 12).

**Table 12:** Outcomes of PRT (this study) and prospective studies of IMRT PRT: proton radiotherapy; IMRT: intensity-modulated radiotherapy; N/A: not available; yr: year

Outcome		PRT (this study)	RTOG-1016 [37]		NRG-HN002 [38]	
			IMRT + cisplatin	IMRT + cetuximab	IMRT + cisplatin	IMRT
Acute toxicity	Dysphagia (grade 3+)	12.8%	37.4%	32%	17.8%	7.5%
	Mucositis (grade 3+)	31.7%	41.5%	46.2%	21.1%	21.1%
	Weight loss (grade 3)	1.4%	7.8%	5.8%	5.9%	3.4%
	Xerostomia (grade 2+)	12.8%	49.7%	53.6%	51.3%	45.6%
	Dermatitis (grade 3+)	18.6%	8%	12.4%	2.6%	5.4%
Late toxicity	Xerostomia (grade 2-3)	19.2%	32.1%	33.6%	26%	19.4%
	Dysphagia (grade 3+)	1.6%	4.4%	6.1%	N/A	N/A
Oncologic outcomes	Overall survival	2 yr: 98.2%; 3 yr: 96.1%	5 yr: 84.6%	5 yr: 77.9%	2 yr: 96.7%	2 yr: 97.3%
	Progression-free survival	2 yr: 92.7%; 3 yr: 86.1%	5 yr: 78.4%	5 yr: 67.3%	2 yr: 90.5%	2 yr: 87.6%
	Locoregional failure	Crude: 2.1%	Crude: 5.7%	Crude: 11.7%	2 yr: 3.3%	2 yr: 9.5%
	Distant failure	Crude: 5.9%	Crude: 7.6%	Crude: 10.7%	2 yr: 4%	2 yr: 2.1%

Our analysis also further defined the toxicity profile associated with PRT. Our finding that acute feeding tube use was lower (2.1-fold) following PRT than IMRT is consistent with earlier reports on intensity-modulated proton therapy (IMPT) for patients with OPCs as well as preliminary results of a prospective trial comparing IMPT and IMRT [36,39]. After six months, however, this difference was no longer significant. Among prospective studies of patients with HPV-associated OPC treated with IMRT and concurrent cisplatin, rates of feeding tube use have decreased in the more recent treatment era: in RTOG-1016, the crude rates of feeding tube use one, six, and 12 months after treatment were 51%, 17%, and 9%, respectively, while in NRG-HN002, they were 22%, 2.8%, and 3.4%, respectively [37,38]. This may be related to multiple factors including reduced dose of RT, improved methods of supportive care before and after treatment, increased integration of dieticians and speech-language pathologists during treatment, and decreased utilization of prophylactic feeding tubes [40].

When compared to prospective studies using IMRT [37,38], adverse event rates associated with PRT appear favorable (Table 12). For example, relative to patients treated with IMRT enrolled on RTOG-1016 and NRG-HN002, patients treated with PRT appear to have similar rates of acute grade 3 or more dysphagia (PRT: 12.8%, RTOG-1016: 32-37.4%, NRG-HN002: 7.5-17.8%) and grade 3 or more mucositis (PRT: 31.7%, RTOG-1016: 41.5-46.2%, NRG-HN002: 21.1%). While rates of acute grade 3 weight loss (PRT: 1.4%, RTOG-1016: 5.8-7.8%, NRG-HN002: 3.4-5.9%) and grade 2 or more xerostomia (PRT: 12.8%, RTOG-1016: 49.7-53.6%, NRG-HN002: 45.6-51.3%) were numerically lower in with PRT compared to IMRT, this should be interpreted with caution as this is not a statistical comparison. Theoretically, lower rates of xerostomia and weight loss could contribute to the lower rates of acute feeding tube use seen with PRT. In terms of late adverse events, rates of grade 2-3 xerostomia (PRT: 19.2%, RTOG-1016: 32.1-33.6%, NRG-HN002: 19.4-26%) appear to be similar between PRT and IMRT, while grade 3 or more dysphagia may be less frequent after PRT (PRT: 1.6%, RTOG-1016: 4.4-6.1%, NRG-HN002: N/A). However, acute dermatitis (grade 3 or more) appears to be more frequent with PRT than IMRT (PRT: 18.6%, RTOG-1016: 8-12.4%, NRG-HN002: 2.6-5.4%). This finding is consistent with prior reports of PRT for other disease sites [41].

In addition to clinician-rated adverse events, this study also provides insight into PROs associated with PRT. PRO measurements are critical to toxicity comparisons between IMRT and PRT: they can reveal aspects of the patient experience that are not apparent in clinician-based assessments, and they can vary by RT technique [42,43]. With PRT, the impact of treatment on PRO quality measures is greatest toward the end of treatment, gradually lessens, and plateaus by 12 months after treatment. These kinetics are similar to those reported in studies of patients treated with IMRT [44,45].

While we could not quantitatively compare PROs between PRT and IMRT, the finding that xerostomia was numerically lower with PRT is not surprising: these symptoms are commonly reported across multiple instruments [46,47], and prior studies have demonstrated that more conformal RT techniques can improve patient-reported xerostomia [48]. The heterogeneity of PRO measures used in these studies highlights the need for the standardization of these measures to improve the interpretability and generalizability of these measures across future studies.

Outcomes reported in this study provide additional insight into the ongoing randomized trials of PRT versus IMRT for patients with OPCs. For example, the primary outcome of the phase III trial sponsored by MD Anderson Cancer Center (NCT01893307) compares PRT and IMRT on the basis of three-year PFS [7]. In this trial, IMRT is assumed to have a three-year PFS of 80%, and PRT will be considered non-inferior if the three-year PFS is within 9 percentage points (hazard ratio (HR) 1.535). Initial results of this trial found that compared to IMRT, PRT was associated with a non-inferior PFS (HR 0.87 on intention-to-treat analysis) and reduced rates of feeding tube dependence (42% versus 28%, respectively) [36]. While our analysis supports both of these findings, the absolute difference in acute feeding tube use between IMRT and PRT was lower in our study (9.6%) compared to this trial (14%).

Since non-inferiority of efficacy may not justify the widespread use of PRT for OPC, an additional understanding of its potential benefit on acute and late RT toxicities is required. The TORPEdO trial (ISRCTN 16424014) compares PRT and IMRT on the basis of patient- and clinician-rated late toxicities [8]. In this study, the co-primary endpoints are the following: (1) the physical composite score of the University of Washington Quality of Life Questionnaire (UW-QoL v4.0), which assesses patients' perceptions on chewing, swallowing, speech, taste, saliva, and appearance, and (2) rates of feeding tube use or CTCAE grade 3 weight loss at 12 months post-RT [8]. The trial is powered to detect an 8-point difference in UW-QoL physical composite score and an 18% difference in late feeding tube use or grade 3 weight loss (25% with IMRT versus 7% with PRT). Studies included in our analysis found that PRT was associated with improved patient-reported dry mouth and xerostomia at 12 months, but not taste, chewing, or swallowing [14,17,24]. Furthermore, although our study did not include weight loss outcomes, PRT and IMRT were associated with similar rates of late feeding tube use. Taken together, these findings suggest that the clinical benefit of PRT assumed by this trial may be smaller than anticipated.

Similar to TORPEdO, the Danish Head and Neck Cancer Group (DAHANCA) 35 (NCT04607694) trial consists of two companion trials (DAHANCA35D and DAHANCA35X) and examines late toxicities after PRT [35]. Patients are enrolled in either trial based on whether normal tissue complication probability (NTCP) models demonstrate at least a 5% improvement in dysphagia or xerostomia using PRT compared to IMRT. DAHANCA35D assesses the rate of clinician-rated dysphagia (grade 2 or more) at six months post-RT and is powered to detect a 12% reduction in dysphagia between PRT and IMRT. In this study, PRT is assumed to have a late dysphagia rate of 16%. This is similar to our study (pooled rate of late grade 2+ dysphagia with PRT, 11.8%) and is lower than the rate of late grade 2+ dysphagia seen on DAHANCA19 (28%). DAHANCA35X, on the other hand, assesses late xerostomia at six months post-RT using the European Organization for Research and Treatment of Cancer Quality of Life Questionnaire-Head and Neck 35 (EORTC QLQ-HN35) instrument. It is designed to detect an 8% reduction in grade 4 xerostomia between IMRT and PRT (10% versus 2%, respectively). In our analysis, one study [17] compared PRT and IMRT using the same instrument and found that the differences in xerostomia or sticky saliva at six months post-treatment were not significant. When examining rates of clinician-rated xerostomia in our study and RTOG-1016, PRT and IMRT appear to have similar rates of late grade 3 xerostomia (1.1% and 1-2%, respectively) [37].

Overall, our study had several strengths. First, this is one of the largest analyses of outcomes following PRT for the upfront management of OPCs. Patients included in this study were treated at academic centers using modern PRT techniques. This analysis was designed prospectively and used appropriate statistics, and confounding due to overlapping patient populations was specifically addressed. This study does, however, have some limitations. Because it is a study-level meta-analysis, detailed comparisons between subgroups were not possible. As a result, we were not able to compare toxicity outcomes by radiation dose (e.g., 60 Gy versus 70 Gy) or by treatment intent (definitive versus adjuvant). Given that multiple studies came from the same institution, the total number of included patients likely overestimates the unique number of patients. Additionally, some outcomes (particularly PROs) were too heterogeneously reported for pooled statistical analyses. As a result, we were unable to assess the impact of different risk factors (e.g., HPV status) on outcomes, and direct comparisons between PRT and IMRT were limited. This highlights the need for consistent reporting standards in institutional series. 

Ultimately, these data further inform the risk-benefit analysis radiation oncologist must make when selecting between radiation treatment modalities. These findings also allow for more comprehensive decision-making for patients, who must weigh the potential benefits of PRT against its potential financial costs and logistical/access barriers.

## Conclusions

For patients with OPCs, PRT is associated with a favorable toxicity profile and high early rates of OS and PFS. Compared to IMRT, PRT is associated with lower rates of acute (but not late) feeding tube use. While awaiting the final results of ongoing randomized trials, this study provides additional evidence regarding the efficacy and toxicity of PRT for the treatment of OPC. 
